# The Association between Music Listening at Home and Subjective Well-Being

**DOI:** 10.3390/bs14090767

**Published:** 2024-09-02

**Authors:** Xin Shan, Yan Zhang, Jie Deng, Haixia Ma, Xiaoxi Hu

**Affiliations:** 1 College of Music, Sangmyung University, Seoul 03016, Republic of Korea; 202134033@sangmyung.kr (X.S.); 201932049@sangmyung.kr (Y.Z.); 2 School of Psychology, Sichuan Normal University, Chengdu 610066, China; 20222093004@stu.sicnu.edu.cn; 3 College of Music, Kookmin University, Seoul 02707, Republic of Korea; 4 State Key Laboratory of Advanced Rail Autonomous Operation, Beijing Jiaotong University, Beijing 100044, China

**Keywords:** subjective well-being, frequency of music listening, mental health, ordered probit model, OLS model

## Abstract

This study examines the association between listening to music at home and subjective well-being, using data from 14,162 respondents in the China General Social Surveys conducted in 2015, 2017, and 2021. Among the respondents, the average happiness score was 3.958. Regression analyzes indicate that frequent music listening is significantly associated with higher happiness levels, with coefficients of 0.384 in the baseline model and 0.570 in the model with control variables. Robustness checks performed across different models support these findings. Instrumental variable analysis, using Mandarin proficiency, yielded a coefficient of 0.212, indicating a robust association despite a slight reduction in magnitude. Heterogeneity analyzes showed consistent associations across genders, religious beliefs, and regions, with slightly stronger associations observed for females and non-religious individuals. Mediation analysis identified mental health and class identity as significant mediators, contributing to a total association of 0.146. These results highlight the positive correlation between music listening and well-being, suggesting the potential value of integrating music resources into well-being strategies.

## 1. Introduction

Subjective well-being is an essential indicator of quality of life. With economic development and rising living standards, people’s pursuit of spiritual fulfillment has become increasingly prominent, making well-being a key area of interest in modern society [1,2,3,4,5,6]. As a widespread cultural phenomenon, music not only serves as a form of recreation but is also associated with psychological regulation, emotional expression, and cultural identity [2,3,7,8,9]. Music is widely used in various cultural and social contexts, such as music therapy to alleviate psychological stress [10], music education to enhance cultural literacy [11], and in social settings to strengthen interpersonal relationships [12]. As a non-pharmacological treatment, music therapy has been extensively utilized in the intervention of various mental health issues [2,3,5,9,13,14].

Listening to music at home, due to its convenience and personalization, has become an integral part of daily life for many individuals. People can freely choose their preferred types of music, allowing for a personalized music experience that enhances subjective well-being. Additionally, listening to music may promote interaction and communication among family members, enhance family harmony, and is associated with higher levels of overall happiness [15]. People who frequently listen to music generally report recovering more quickly from negative emotions and experiencing more joy and satisfaction in their daily lives [16].

Although existing studies have explored the relationship between music and mental health and well-being [9], empirical research on the relationship between the frequency of listening to music at home and subjective well-being remains relatively scarce. To address this research gap, this study aims to analyze the association between the frequency of listening to music and subjective well-being, providing deeper insights into the value and role of music in daily life. The current study is based on the theory of emotional regulation, exploring how music is related to subjective well-being through emotional regulation. We hypothesize that a higher frequency of listening to music at home is correlated with stronger subjective well-being. Additionally, we will examine gender differences and the associations between mental health and the outcomes.

The data for this research was sourced from the 2021 China General Social Survey (CGSS), which has high relevance and reliability. Detailed information on the sample size and selection process will be provided to ensure the representativeness and credibility of the research results.

The core variables used in this study include the frequency of listening to music and subjective well-being. The frequency of listening to music will be measured using specific frequency questions from the questionnaire, while subjective well-being will be assessed through a self-rating scale. The measurement methods and relevance of these variables will then be thoroughly explained. To mitigate potential confounding factors and strengthen the internal validity of the study, we will control for variables such as gender, age, and education. These variables are selected based on clear theoretical justifications, contributing to the accuracy of the research results.

Through in-depth research, we hope to provide scientific evidence and practical guidance on using music to enhance personal well-being, thereby potentially promoting mental health and improving the quality of life. The implications of our study results are discussed concerning not only the enhancement of individual well-being but also possible broader application of music at the social level, such as enhancing collective well-being through community music activities or promoting mental health knowledge in schools through music education. Ultimately, we hope that the results of this study will provide valuable references for policymakers, educators, and mental health professionals, contributing to the achievement of a healthier and happier society.

## 2. Literature Review and Hypothesis

### 2.1. Individual Well-Being

Individual well-being generally refers to people’s subjective assessment of their quality of life, encompassing their emotional reactions and life satisfaction. Subjective well-being is an important research area in psychology and social sciences because it reflects individuals’ overall quality of life and happiness. Methods for measuring subjective well-being include self-report questionnaires and interviews, which can capture individuals’ emotional states and overall life satisfaction. Odermatt and Stutzer have explored the use of subjective well-being measures in public policy, emphasizing their importance for policy development [17]. Lucas et al. identified and corrected four common misconceptions about subjective well-being [18]. Alatartseva and Barysheva categorized individual well-being into objective and subjective aspects, highlighting the importance of assessing subjective well-being in understanding an individual’s quality of life [19]. Diener et al. argued that subjective well-being is a core component of overall well-being and directly affects an individual’s mental health and life satisfaction [20].

Tay and Diener explored the relationship between need fulfillment and subjective well-being and found that the level of need fulfillment is associated with an individual’s life satisfaction [21]. Dubé et al. examined individuals’ perceptions of their well-being and found that people were more likely to attribute their well-being to internal factors than to external circumstances [22]. Stutzer and Frey reviewed the progress of individual subjective well-being research in economics, noting its potential to inform economic policy-making [6]. Kennedy et al. discussed the challenges in measuring subjective well-being and provided recommendations for addressing these challenges [23]. Pavot and Diener studied subjective well-being in adulthood and found that age, personality, and environmental factors are significantly associated with well-being [24]. Ray investigated the relationship between work environment and subjective well-being, finding a significant correlation between job satisfaction and overall well-being [25].

Diener et al. explored the associations between culture, personality, and subjective well-being, finding significant differences in affective and cognitive ratings of well-being among individuals from different cultural backgrounds [26]. In addition, in 2018, they reviewed recent advances in subjective well-being research, proposing a variety of new research methods and measurement tools [27]. Kapteyn et al. examined the associations between different dimensions of subjective well-being, including affective experience, cognitive appraisal, and daily activities [28]. These studies provide a rich perspective for understanding the multifaceted nature of subjective well-being. By analyzing research findings from different fields and perspectives, a more comprehensive understanding of the components and factors associated with subjective well-being can be achieved, thereby offering theoretical foundations and empirical insights for the development and refinement of related policies.

### 2.2. The Association between Listening to Music and Individual Well-Being

Numerous studies have explored and investigated the associations between listening to music and individual well-being. For example, Hu et al. showed that college students use music to promote learning and well-being [29]. Laukka concluded that music was positively associated with the mental health of older people [30]. Krause et al. reported that music helps individuals cope with daily stress [31], while Linnemann et al. noted the association between listening to music and reduced stress in different social environments [32]. Majeed et al. specifically investigated the associations between bedtime music listening and subjective sleep quality, as well as next-morning well-being, revealing that music listening before sleep is associated with better sleep quality and enhanced well-being the following morning [33]. MacDonald reviewed the positive associations between informal music listening and health and well-being [9]. Dingle et al. examined the psychological mechanisms by which musical activity affects health and well-being [34]. Biley reviewed the associations between music listening as a nursing intervention and patient well-being [35]. Bonus analyzed the associations between listening to nostalgic music and well-being [36]. Skånland emphasized the role of MP3 players in everyday music listening and emotion regulation [37]. Loepthien and Leipold investigated the experience of heart flow in music performance and listening and its relation to subjective well-being [38]. Miranda and Gaudreau emphasized the association between music listening and adolescents’ emotional well-being [39]. These studies suggest that music listening significantly enhances individual well-being through multiple pathways.

These studies indicate that individual well-being is associated with economic status, social relationships, health, psychological attributes, and life environment. Research has consistently linked music listening with enhanced well-being, showing that it positively affects mental health, stress management, and emotional regulation.

Despite these positive findings, there is a notable gap in the literature concerning the frequency of self-listening to music at home. This aspect is crucial because the regularity of music listening at home could be associated with an individual’s emotional state and overall life satisfaction. Given the vital role music plays in emotional expression and regulation, examining the relationship between listening frequency and well-being could offer valuable insights into its potential therapeutic benefits.

In this study, we incorporate the frequency of listening to music at home into the framework for analyzing individual well-being, exploring its association with subjective well-being. Based on the previous discussion, we propose the following hypothesis:

**Hypothesis 1:**  *There is a significant positive association between the frequency of listening to music at home and subjective well-being.*

Listening to music is a common and easily accessible form of entertainment that provides positive emotional experiences and psychological satisfaction. Frequent music listening at home may be associated with increased emotional stability and well-being, as music helps individuals relax, reduce stress, and provides pleasurable feelings in everyday life. Studies have shown that people who listen to music tend to report higher life satisfaction and better subjective well-being [31]. This is because music not only regulates mood but also promotes mental health and emotional expression. Therefore, it is hypothesized that more frequent music listening at home is associated with greater subjective well-being. Based on these arguments, the following hypothesis is proposed:

**Hypothesis 2:**  *The frequency of listening to music at home is significantly associated with individuals’ subjective well-being through the mediating relationships of mental health and enhanced class identity.*

Listening to music is positively associated with mental health, which in turn is significantly associated with the individual’s subjective well-being [9]. Specifically, music serves functions such as relaxation, stress reduction, and emotional enhancement. Regularly listening to music at home can be associated with better personal mental health by reducing symptoms of anxiety and depression, and by enhancing emotional regulation [3]. Moreover, music can also enhance the individual’s cultural taste and social interaction abilities, indirectly improving class identity and thereby increasing subjective well-being. Mental health and class identity serve as two mediating variables, improving psychological states and enhancing class identity, thereby being associated with the individual’s subjective well-being. Therefore, we hypothesize that the positive association between the frequency of listening to music at home and subjective well-being is mediated through better mental health and stronger class identity.

## 3. Data, Variables, and Modeling Methods

### 3.1. Data Sources and Description of Variables

#### 3.1.1. Data Source

This study utilizes data from the Chinese General Social Survey (CGSS), a nationally representative survey that employs multi-stage stratified sampling techniques. The CGSS collects data annually from urban and rural households in China, providing socio-economic trends and the relationship between quality of life and the socio-economic structure of the country. The survey consists of two phases from 2003 to 2019. For this empirical analysis, we used data from the second phase due to its detailed focus on well-being.

The CGSS employs proportionate multi-stage stratified sampling techniques. Initially, data were collected from 100 districts (counties) and five major cities: Shanghai, Guangzhou, Beijing, Shenzhen, and Tianjin. In the second stage, four village committees or neighborhoods were randomly selected in each district. In the final stage, approximately 25 households were randomly chosen from each village committee or neighborhood, with one individual from each household selected for interviews. In the five major cities mentioned, 80 committees were established, resulting in around 480 neighborhood committees being interviewed nationwide, with a response rate exceeding 70%.

The CGSS is particularly suited for our analysis, as it provides comprehensive data on respondents’ employment status, income levels, education, health status, social interactions, and other socio-economic characteristics. The second phase of the CGSS data, spanning from 2010 to 2019, is publicly accessible. To ensure a sufficiently large analytical sample, we combined data from the CGSS surveys conducted in 2015, 2017, and 2021. After a rigorous review and the removal of invalid and abnormal data, we obtained 14,162 valid samples, ensuring the accuracy and representativeness of our analysis.

#### 3.1.2. Dependent Variable

The dependent variable in this study is happiness. Referring to Yin and Liu [40], this variable is derived from the CGSS questionnaire item: “Overall, how happy do you think your life is?” The valid responses are five options: “Very unhappy”, “Somewhat unhappy”, “Not very happy”, “Somewhat happy”, and “Very happy”. Each response is assigned a value from 1 to 5, with higher values indicating higher levels of happiness.

#### 3.1.3. Independent Variable

The frequency of listening to music at home is the independent variable. This variable originates from a specific question in the CGSS: “How often do you usually listen to music at home?” The responses are rated on a 5-point scale, ranging from 1 (never) to 5 (very frequently). The minimum and maximum values of 1 and 5 represent the entire range from not at all frequent to very frequent, respectively.

#### 3.1.4. Control Variables

Considering the research topic, the factors influencing happiness, and the conclusions and recommendations from previous studies, the control variables in this study include gender, age, religion, ethnicity, self-rated health, marital status, social activities, and perception of social fairness.

Gender is included as a binary variable, with 0 representing male and 1 representing female. Age is calculated as the difference between the survey date and the participant’s date of birth, providing a continuous measure of age. Ethnicity is categorized into two groups: 0 for Han ethnicity and 1 for minority ethnic groups. Religious belief is also a binary variable, where 0 indicates no religious belief and 1 indicates the presence of religious belief.

Self-rated health is assessed on a scale from 1 to 5, with higher values indicating better self-rated health. Marital status is classified into 0 for unmarried and 1 for married individuals. Social activities are measured on a scale from 1 to 7, evaluating the extent of engagement in social activities. Finally, perceived social fairness is evaluated on a scale from 1 to 5, reflecting participants’ perceptions of fairness in social contexts.

By including these control variables, the study aims to account for various factors that might be associated with individual well-being, thus providing a clearer understanding of the relationship between music listening and happiness [4,5,6].

#### 3.1.5. Mediating Variable

Referring to previous studies [41], this study primarily considers two mediating variables—mental health and class identity—and examines the relationship between listening to music at home and happiness. Mental health is measured through the Chinese General Social Survey (CGSS) question: “In the past four weeks, how often have you felt depressed or downhearted?” with the response options: always, often, sometimes, and rarely. The options for class identity are ‘upper class’, ‘upper middle class’, ‘middle class’, ‘lower middle class’, and ‘lower class.

### 3.2. Modeling and Methodology

#### 3.2.1. Ordered Probit Model

To explore the relationship between listening to music at home and individual subjective well-being, an ordered probit model is first used for preliminary estimation. The ordered probit model is a statistical method specifically designed for situations where the dependent variable is an ordinal categorical variable. The model assumes the existence of an underlying continuous variable that explains the observed ordinal discrete outcomes. In the model, independent variables are associated with this latent variable, and based on its values, the observed outcomes are categorized into different levels. By estimating the association between the independent variables and the latent variable, as well as the thresholds between categories, the model can predict and explain the direction and magnitude of the relationship between the independent variables and the dependent variable. The model is specified as follows:

(1) $$happiness_{i}=\alpha_{1}+\beta_{1}music_{i}+\delta_{1}X_{i}+\epsilon_{i}$$

where $happiness_{i}$ represents the happiness of the i-th respondent, $music_{i}$ is the frequency of listening to music at home for the i-th respondent, $X_{i}$ refers to a series of control variables, and $\beta_{1}$ is the coefficient to be estimated, reflecting the size and direction of the association between the frequency of listening to music and subjective well-being.

#### 3.2.2. Robustness Checks

To ensure the robustness of the regression results, we referred to the study by Ferrer-i-Carbonell and Frijters [42]. They pointed out that as long as the regression equation is correctly specified, the empirical conclusions will not be fundamentally affected by whether residents’ happiness is treated as a continuous variable in Ordinary Least Squares (OLS) regression or as an ordinal variable in ordered Probit or ordered Logit regression.

The ordered Probit and Logit models are specifically designed for situations where the dependent variable is ordinal, meaning it has a natural order but the intervals between categories are not necessarily equal. These models assume the existence of an underlying continuous latent variable that is associated with the observed ordinal outcomes. By estimating the thresholds between categories and the relationship between independent variables and the latent variable, these models provide a way to interpret the direction and significance of associations in a manner consistent with OLS, although the coefficients themselves may differ slightly in value.

Specifically, the direction and significance of the coefficients remain highly consistent across these methods, with only minor differences in the coefficient values. Therefore, in this study, we conducted both OLS and ordered Logit regression analyzes to validate the robustness of the results.

#### 3.2.3. Instrumental Variable Model

To reduce potential bias in the estimated regression coefficients due to omitted variables, this study incorporates a broad set of individual-level information. However, there may still be a potential bidirectional relationship between the frequency of listening to music and its association with subjective well-being. Therefore, following the approach of Zi Shu Rong [43], this study considers using the instrumental variable (IV) method to address endogeneity concerns. The instrumental variable method is a statistical technique used when the independent variable of interest is correlated with the error term in a regression model, leading to biased and inconsistent estimates. By introducing an external variable—known as an instrumental variable—that is correlated with the endogenous independent variable but uncorrelated with the error term, the IV method allows for a more accurate estimation of the relationship between variables.

In this study, the instrumental variable introduced is the individual’s self-perception of their ability to understand Mandarin. Since language is a unique cultural phenomenon, understanding and engaging with culture requires corresponding language proficiency. Thus, the self-perception of understanding Mandarin is chosen as the instrumental variable. The ability to understand Mandarin is divided into five levels: “completely do not understand”, “poor”, “average”, “good”, and “very good”, with values assigned from 1 to 5, respectively.

#### 3.2.4. Mediation Analysis Model

Mediation analysis is used to study how an independent variable is associated with a dependent variable indirectly through a mediator. By comparing the association between the independent variable and the dependent variable before and after introducing the mediator, if the association between the independent variable and the dependent variable diminishes or changes when the mediator is included, and the mediator itself shows a significant relationship with the dependent variable, it indicates the presence of a mediation relationship. Following existing research [44], to test for potential mediation relationship, the following models are constructed based on Model (1):

(2) $$Mediating_{i}=\alpha_{2}+\beta_{2}music_{i}+\delta_{2}X_{i}+\epsilon_{i}$$

(3) $$happiness_{i}=\alpha_{3}+\beta_{3}music_{i}+\gamma Mediating_{i}+\delta_{3}X_{i}+\epsilon_{i}$$

where $Mediating_{i}$ refers to the mediator variable.

The first step is to test the significance of $\beta_{1},\beta_{2},\beta_{3}$. If $\beta_{1}$ is significant, it indicates that the frequency of listening to music is significantly associated with the mediating variable; if $\beta_{3}$ is significant, it indicates that the frequency of listening to music is significantly associated with happiness; if γ is significant, it indicates that the mediating variable is significantly associated with happiness. Finally, if $\beta_{3}$ decreases in significance or becomes insignificant after introducing the mediating variable, it indicates the presence of a mediation relationship.

## 4. Associations between the Frequency of Music Listening at Home and Individual Subjective Well-Being

### 4.1. Descriptive Statistics

Table 1 presents the descriptive statistics for each variable. The average happiness score among respondents is 3.958. Gender distribution is relatively balanced, with males comprising 48.1% of the sample. In terms of ethnicity, Han Chinese respondents constitute 93.3%. The average score for religious belief is 0.082, indicating a low proportion of respondents with religious beliefs. The average self-rated health score is 3.695, suggesting a generally positive perception of health status. Married respondents account for 77% of the sample. The average score for perceived social fairness is 3.433, reflecting variability in respondents’ perceptions. The average score for social activities is 4.156, indicating significant differences in the frequency of social activities among respondents.

### 4.2. Benchmark Regression

Table 2 presents the results of the baseline regression models. Model 1 uses the “frequency of listening to music at home” as the independent variable to explore its association with happiness. The results indicate that the “frequency of listening to music at home” is significantly associated with happiness, with a regression coefficient of 0.384; the association is statistically significant (*p* < 0.01 and ≥0.10). Model 2 extends Model 1 by introducing additional control variables. The regression coefficient for “frequency of listening to music at home” increases to 0.570, and the association remains statistically significant (*p* < 0.01).

Additionally, Model 2 reveals several significant predictors of happiness. Gender shows a significant negative association with happiness, suggesting lower happiness levels among males compared to females. Ethnicity exhibits a significant positive association with happiness. Respondents with religious beliefs report significantly higher levels of happiness, while those with higher self-rated health status also exhibit increased happiness. Married respondents demonstrate significantly higher happiness levels than unmarried individuals. Perceived social fairness and frequency of social activities are positively associated with happiness. All these differences are statistically significant (*p* < 0.01 and ≥0.10), indicating that even after adjusting for multiple control variables, the “frequency of listening to music at home” remains significantly associated with happiness. The findings support Hypothesis 1.

### 4.3. Robust Regression

This study employed both OLS and ordered logit models (see Table 3) to conduct robustness checks on the research results, ensuring the rigor of the analysis and the reliability of the findings. In addition to these methods, we applied a dummy variable approach to further explore the association between music listening at home and well-being. We reclassified the original scale by grouping scores of 1–3 as 0 (indicating low frequency) and 4–5 as 1 (indicating high frequency). The categorization was used to provide a clearer exploration of the relationship between music and well-being. A comparison between Table 2 and Table 3 shows that, regardless of whether OLS or ordered logit regression is used, the direction and significance of the coefficients for all explanatory variables remain consistent, with only slight differences in coefficient magnitudes. This consistency further validates the robustness of the observed relationship between the frequency of music listening at home and well-being, indicating that the conclusions drawn from different statistical methods are both consistent and reliable.

### 4.4. Empirical Results of the Instrumental Variable (IV) Method

Table 4 summarizes the two-stage least squares (2SLS) estimation results using Mandarin proficiency as an instrumental variable for the frequency of listening to music at home. Column (1) presents the baseline regression results from the Ordered Probit model, Column (2) details the first-stage regression results of the instrumental variable, and Column (3) describes the second-stage regression results.

In the first-stage regression, a significant correlation is observed between Mandarin proficiency and the frequency of listening to music at home, with a coefficient of 0.3687 and a significance level of 0.001. The Sargan statistic for the overidentification test is 0.000, indicating that the model is exactly identified. The Cragg-Donald Wald F statistic for the weak instrument test is 1081.25, which exceeds all Stock-Yogo critical values, confirming the strength of the instrumental variable. The Anderson canonical correlation LM statistic for the underidentification test is 1005.15, with a *p*-value of 0.000, indicating that the model is correctly identified.

In the second-stage regression, when estimated with the instrumental variable, a significant positive association is found between the frequency of listening to music at home and happiness, with a coefficient of 0.2118 and a significance level of 0.001. However, the estimated coefficient is smaller than that observed in the baseline regression, suggesting that endogeneity may have previously led to an overestimation of the positive association between music listening and happiness.

### 4.5. Heterogeneity Analysis of the Association between the Frequency of Listening to Music at Home and Individuals’ Subjective Well-Being

Table 5 presents the heterogeneous associations between the frequency of listening to music at home and happiness, exploring variations across dimensions such as gender, religious belief, and regional distribution. The regression coefficient for the frequency of listening to music at home and happiness is 0.587 for females and 0.560 for males. For religious belief, the coefficient is 0.574 for those without religious beliefs and 0.516 for those with religious beliefs. Regarding regional distribution, the coefficient is 0.571 for residents in non-eastern regions and 0.558 for residents in eastern regions. All these results have *p*-values less than 0.01, indicating that the association between the frequency of listening to music at home and happiness is statistically significant.

The findings in Table 5 indicate that the positive association between the frequency of listening to music at home and happiness is consistent across different genders, religious beliefs, and regions, although the magnitude of the association varies. Specifically, the association with happiness is slightly stronger for females and individuals without religious beliefs compared to males and those with religious beliefs, respectively. Additionally, residents in non-eastern regions show a slightly stronger association with happiness compared to those in eastern regions. These findings suggest that while the frequency of listening to music at home generally and robustly associates with enhanced happiness, the strength of this association varies among different groups.

### 4.6. Analysis of the Mediation Relationship in the Association between the Frequency of Listening to Music at Home and Individuals’ Subjective Well-Being

Referring to the theoretical assumptions discussed earlier, we further examined the roles of “mental health” and “class identity” as mediating variables.

Table 6 presents the mediation analysis of the association between the frequency of listening to music at home and happiness, with mental health as a mediator. Model 1 shows a significant positive association between the frequency of listening to music at home and mental health (coefficient = 0.205, *p* < 0.01). In Model 2, after including mental health, the association between the frequency of listening to music at home and happiness is 0.528 (*p* < 0.01), while the association of mental health with happiness is 0.126 (*p* < 0.01), indicating that mental health significantly mediates this association. To further validate these mediation relationships, we employed the bootstrap method with 5000 random samples. The results reveal a direct association of 0.131 between the frequency of listening to music at home and subjective happiness, with an indirect association through mental health of 0.014, resulting in a total association of 0.146. The bootstrap analysis (with indirect association ranging from 0.013 to 0.016) confirms the significance of mental health as a mediator.

Table 7 presents the analysis of class identity as a mediating variable. The results reveal a significant positive association between the frequency of listening to music at home and class identity (coefficient = 0.184, *p* < 0.01). When class identity is included in the model, the coefficient for the frequency of listening to music at home in relation to happiness is 0.539 (*p* < 0.01), and the coefficient for class identity in relation to happiness is 0.456 (*p* < 0.01). This indicates that class identity also plays a significant role in this association. Using the bootstrap method, the results show that the indirect association through class identity is 0.018, with a total association of 0.146. The bootstrap inspection results (indirect association of class identity ranging from 0.016 to 0.020) further confirm the significance of class identity as a mediator.

The analysis results demonstrate that the frequency of listening to music at home is positively associated with higher happiness. Additionally, there is an indirect association between the frequency of listening to music at home and higher happiness through changes in mental health and class identity. These findings underscore the significant role of the frequency of listening to music at home in enhancing happiness and support the research Hypothesis 2.

## 5. Discussion

The central finding of this study is that there is a significant positive correlation between the frequency of listening to music at home and subjective well-being, consistent with previous research findings. Boer and Abubakar’s study showed that the prevalence of music in the home environment is positively associated with young people’s social cohesion and emotional well-being [45]. Similarly, Cho and Ilari found that the use of music as part of family activities is significantly associated with enhanced psychological well-being and life satisfaction for both children and mothers [46]. These findings support the notion that listening to music at home is associated with enhanced individual subjective well-being, underscoring the importance of music in family and everyday life.

This study further confirms that the frequency of listening to music at home remains significantly associated with subjective well-being even after controlling for variables such as gender, age, marital status, education level, health status, sense of social equity, and personal socioeconomic status. This aligns with the findings of Grau-Sánchez et al., which concluded that the frequency of musical activities is significantly associated with the emotional well-being among older adults and that this association remained significant after controlling for marital status, level of education, and socioeconomic status [47]. Age may affect the correlation between music and well-being, as changes in life circumstances—such as increased susceptibility to stressors like health issues and social isolation—might alter how music relates to emotional expression and resilience.

The mechanism analysis results indicate that mental health and social status mediate the association between the frequency of listening to music and well-being. Listening to music at home is correlated with well-being through its association with reduced stress, mood changes, and a greater sense of belonging and social connection via shared musical experiences. This is consistent with the findings of Gustavson and Gregory [3,9], who noted the association between music and mental health as well as social status, highlighting music’s role in well-being. Unlike previous research, this study explores this association within the adult population in China for the first time, revealing how music correlates with well-being through its association with social status. Our findings support the role of music in emotional regulation and the mediating role of self-rated mental health and social status.

## 6. Recommandations and Limitations

Based on the research results, we propose the following policy recommendations: First, governments and relevant institutions should implement policies that would enhance the mental health and well-being of older adults through music. Specific measures could include organizing regular concerts, singing groups, and music therapy sessions in community centers, nursing homes, and other venues where older adults gather. These activities may be associated with reduced stress, changes in mood, and enhanced social interactions. Additionally, training professional music therapists and encouraging community volunteers to participate could be associated with a better quality of life for older adults through engagement with music.

Second, schools and universities should utilize music to enhance students’ mental health and well-being and further explore how music can help reduce stress. Academic institutions can incorporate more music classes and offer various musical activities such as bands, choirs, and music creation workshops. Schools should also establish dedicated mental health support services that incorporate music therapy methods. Such integration may help students alleviate stress, enhance mood, and boost self-efficacy through participation in musical activities. By implementing these measures, the potential benefits of music can be better utilized across different age groups to support mental health and social well-being.

Despite the significant findings regarding the association between music listening frequency and subjective well-being, this study has some limitations. First, the data were collected from a cross-sectional survey. Although the sample size is large and representative, the data were collected at a single point in time, making it difficult to reflect long-term trends and changes. Future research could use longitudinal data to gain a more comprehensive understanding of the dynamic relationship between music listening frequency and subjective well-being.

Second, this study mainly relies on self-reported data, and respondents’ subjective judgments may have certain biases. Future research could combine objective tests and experimental data to enhance the reliability of the results. This adjustment could help reduce inherent biases in self-reported data, thereby increasing the reliability of the research findings.

Additionally, this study was conducted only in China, neglecting cross-cultural comparative studies. Different countries and regions may have significant differences in music culture and perceptions of well-being. Future research could extend to other countries and regions to examine the association between music and well-being across diverse cultural contexts.

This study did not explore the associations between different types of music, listening contexts, and well-being, which future research could investigate further. For example, different music types and listening contexts may be associated with varying levels of well-being. Additionally, examining the relationship between music listening frequency and well-being among individuals with different educational backgrounds, occupational types, and socioeconomic statuses could offer more comprehensive insights for policy recommendations.

This study highlights the importance of music education in promoting mental health and enhancing public education by analyzing the relationship between the frequency of listening to music at home and subjective well-being. Future research should extend to other mental health issues and different population characteristics to provide more detailed policy recommendations. These findings will not only enrich the theoretical framework but also provide strong support for mental health education and policy formulation. There is a potential association between enhancing public musical literacy and higher levels of subjective well-being, which may contribute to broader benefits in mental health and social well-being. This association could help address mental health challenges in modern society and support sustainable social development and overall well-being objectives.

## Figures and Tables

**Table 1:** Descriptive statistics of variables.

Variable	Sample Size	Maximum	Minimum	Mean	Standard Deviation	Median	Variance
Happiness	14,162	5	1	3.958	0.58	4	0.336
Listening to Music at Home	14,162	5	1	2.736	1.46	3	2.13
Gender	14,162	1	0	0.481	0.5	0	0.25
Ethnicity	14,162	1	0	0.933	0.25	1	0.063
Religious Belief	14,162	1	0	0.082	0.274	0	0.075
Self-Rated Health	14,162	5	1	3.695	1.008	4	1.015
Marital Status	14,162	1	0	0.77	0.421	1	0.177
Perceived Social Fairness	14,162	5	1	3.433	0.845	4	0.714
Social Activities	14,162	7	1	4.156	1.697	5	2.881

**Table 2:** Association between the frequency of listening to music at home and subjective well-being.

Variable	(1) Happiness	(2) Happiness
Frequency of Listening to Music at Home	0.384 ***	0.570 ***
	(41.40)	(42.08)
Gender		−0.133 ***
		(−4.94)
Ethnicity		0.112 **
		(2.04)
Religious Belief		0.901 ***
		(16.81)
Self-Rated Health		0.288 ***
		(20.97)
Marital Status		0.894 ***
		(26.19)
Perceived Social Fairness		0.984 ***
		(50.29)
Social Activities		0.431 ***
		(39.21)
N	14,162	14,162
R^2^	0.1033	0.4078

Note: ** and *** indicate significance at the 5%, and 10% levels, respectively. The t values are in parentheses.

**Table 3:** Home Music Listening Frequency and Subjective Well-being Robust Regression Analysis.

Variable	Ordered Logit (Happiness)	OLS (Happiness)	Ordered Logit (Happiness2)	OLS (Happiness2)
Frequency of Listening to Music at Home	1.071 ***	0.146 ***		
	(40.89)	(53.10)		
Frequency of Listening to Music at Home2			24.11	0.100 ***
			(0.04)	(32.42)
Gender	−0.253 ***	−0.0343 ***	0.343 **	−0.00109
	(−5.02)	(−4.82)	(2.05)	(−0.39)
Ethnicity	0.234 **	0.0289 **	−0.258	−0.00150
	(2.33)	(2.01)	(−0.81)	(−0.27)
Religious Belief	1.685 ***	0.272 ***	20.11	0.0824 ***
	(17.22)	(20.56)	(0.01)	(15.95)
Self-Rated Health	0.555 ***	0.117 ***	0.640 ***	0.0399 ***
	(21.34)	(32.11)	(8.46)	(28.10)
Marital Status	1.707 ***	0.289 ***	3.752 ***	0.0838 ***
	(26.42)	(32.76)	(15.24)	(24.48)
Perceived Social Fairness	1.882 ***	0.296 ***	3.004 ***	0.0782 ***
	(49.14)	(68.73)	(21.93)	(46.51)
Social Activities	0.805 ***	0.111 ***	0.743 ***	0.0240 ***
	(38.16)	(49.74)	(11.57)	(27.76)
N	14,162	14,162	14,162	14,162
R^2^	0.4083	0.476	0.7662	0.7662

Note: ** and *** indicate significance at the 5%, and 10% levels, respectively. The t values are in parentheses.

**Table 4:** IV Method 2SLS Home Music Listening Frequency and Subjective Well-being.

Variable	Model 1 Ordered Probit (Happiness)	Model 2 First (Happiness)	Model 3 Second (Happiness)
Frequency of Listening to Music at Home	0.570 ***		0.212 ***
	(42.08)		(19.43)
Mandarin Proficiency		0.368 ***	
		(32.76)	
Control Variables	Yes	Yes	Yes
Overidentification Test	0.000		
Weak Identification Test	1081.25 > 16.38		
Underidentification Test	1005.15 ***		

Note: *** indicate significance at the 10% levels. The t values are in parentheses.

**Table 5:** Heterogeneity Analysis Results.

Variable	(1) Female (Happiness)	(2) Male (Happiness)	(3) No Religious Belief (Happiness)	(4) Religious Belief (Happiness)	(5) Non-Eastern (Happiness)	(6) Eastern (Happiness)
Frequency of Listening to Music at Home	0.587 ***	0.560 ***	0.574 ***	0.516 ***	0.571 ***	0.558 ***
	(31.00)	(28.59)	(40.78)	(9.27)	(30.38)	(27.85)
Control Variable	Yes	Yes	Yes	Yes	Yes	Yes
N	7345	6817	13,004	1158	8063	6099
R^2^	0.4298	0.3869	0.4098	0.4059	0.4157	0.3930

Note: *** indicate significance at the 10% levels. The t values are in parentheses.

**Table 6:** Mediation Analysis with Mental Health as Mediator.

Variable	Model1 Mental Health (Happiness)	Model2 Happiness (Happiness)
Frequency of Listening to Music at Home	0.205 ***	0.528 ***
	(27.80)	(38.22)
Mental Health		0.126 ***
		(28.37)
Control Variable	Yes	Yes
N	14,162	14,162
R^2^	0.0905	0.4356
Direct Association		0.131
Indirect Association		0.014
Aggregate Association		0.146
Bootstrap Inspection		0.013–0.016

Note: *** indicate significance at the 10% levels. The t values are in parentheses.

**Table 7:** Mediation Analysis with Class Identity as Mediator.

Variable	Model1 Mental Health (Happiness)	Model2 Happiness (Happiness)
Frequency of Listening to Music at Home	0.184 ***	0.539 **
	(25.28)	(38.83)
Class Identity		0.456 ***
		(22.74)
Control Variable	Yes	Yes
N	14,162	14,162
R^2^	0.226	0.4354
Direct Association		0.128
Indirect Association		0.018
Aggregate Association		0.146
Bootstrap Inspection		0.016–0.02

Note: ** and *** indicate significance at the, 5%, and 10% levels, respectively. The t values are in parentheses.

## Data Availability

General correspondence and requests for source data and materials should be addressed to Haixia Ma. Requests for access to data should be addressed to mahaixia814@kookmin.ac.kr.
